# Preface the 2nd Wakayama symposium: current concepts in ocular cell biology

**DOI:** 10.1186/s12886-015-0156-2

**Published:** 2015-12-17

**Authors:** Shizuya Saika

**Affiliations:** Wakayama Medical University School of Medicine, 811-1 Kimiidera, Wakayama, 641-0012 Japan

It is a great pleasure for me to have a chance to host this opportunity of getting together and discussing our interest, ocular surface cell biology, at Wakayama Medical University, Wakayama, Japan again. The aim of the symposium is promoting international exchange of our research on biology, pathophysiology and clinical issues on cornea and conjunctiva, and also stimulating our research corporation as well as to strengthen our friendship.

Corneal transparency and maintenance of its regular shape are critical for our vision. Therefore, to understand the physiology and pathology of this lovely tissue is quite important for quality of vision and thus of life of people. The topics presented in the symposium covered the important aspects of the cornea and ocular surface cell biology. Besides free paper presentation we had a seven wonderful lectures by distinguished investigators. The topics in them included findings from researchers on dry eye syndrome and related fields, i. e., Meibomian gland (Dr. Jester), goblet cell differentiation (Dr. Liu), ocular surface mucins (Dr. Shirai, et al.) and immunoresponse in dry eye-ocular surface (Dr. Joo). Roles of mesenchymal cells in ocular tissue fibrosis (Dr. Yamanaka, et al.) and regenerative medicine (Dr. Kao) were also to be much noted. Dr. Reinach and his colleagues gave us a wonderful talk on a novel system by TRP ion channel receptors in maintenance of ocular surface homeostasis. I am so proud of the great success of our symposium. I sincerely would like to express my deep thanks to all the attendees. I believe each manuscript by these seven teams provide us significant impacts in the field of ocular surface research.

